# Whole Body MRI at 3T with Quantitative Diffusion Weighted Imaging and Contrast-Enhanced Sequences for the Characterization of Peripheral Lesions in Patients with Neurofibromatosis Type 2 and Schwannomatosis

**DOI:** 10.5402/2013/627932

**Published:** 2013-10-07

**Authors:** Laura M. Fayad, Jaishri Blakeley, Scott Plotkin, Brigitte Widemann, Michael A. Jacobs

**Affiliations:** ^1^The Russell H. Morgan Department of Radiology and Radiological Science, The Johns Hopkins University School of Medicine, Baltimore, MD 21287, USA; ^2^Sidney Kimmel Comprehensive Cancer Center, The Johns Hopkins University School of Medicine, Baltimore, MD 21287, USA; ^3^Department of Orthopaedic Surgery, The Johns Hopkins University School of Medicine, Baltimore, MD 21287, USA; ^4^The Johns Hopkins Hospital Comprehensive Neurofibromatosis Center, Department of Neurology, The Johns Hopkins Hospital, CRB II, Suite 1M16, 1550 Orleans Street, Baltimore, MD 21231, USA; ^5^Department of Neurology and Cancer Center, Massachusetts General Hospital, Boston, MA, USA; ^6^Neurofibromatosis Clinic, Pappas Center for Neuro-Oncology, Massachusetts General Hospital, 55 Fruit Street, YAW 9, Boston, MA 02114, USA; ^7^Center for Cancer Research, National Cancer Institute, Bethesda, MD, USA; ^8^Pharmacology & Experimental Therapeutics Section, Pediatric Oncology Branch, NCI, CCR, Room 1-5750, 10 Center Drive, 10-CRC, MSC 1101, Bethesda, MD 20892, USA

## Abstract

*Purpose*. WB-MRI is mainly used for tumor detection and surveillance. The purpose of this study is to establish the feasibility of WB-MRI at 3T for lesion characterization, with DWI/ADC-mapping and contrast-enhanced sequences, in patients with neurofibromatosis type 2 (NF-2) and schwannomatosis. *Materials and Methods*. At 3T, WB-MRI was performed in 11 subjects (10 NF-2 and 1 schwannomatosis) with STIR, T1, contrast-enhanced T1, and DWI/ADC mapping (*b* = 50, 400, 800 s/mm^2^). Two readers reviewed imaging for the presence and character of peripheral lesions. Lesion size and features (signal intensity, heterogeneity, enhancement characteristics, and ADC values) were recorded. Descriptive statistics were reported. *Results*. Twenty-three lesions were identified, with average size of 4.6 ± 2.8 cm. Lesions were characterized as tumors (21/23) or cysts (2/23) by contrast-enhancement properties (enhancement in tumors, no enhancement in cysts). On T1, tumors were homogeneously isointense (5/21) or hypointense (16/21); on STIR, tumors were hyperintense and homogeneous (10/21) or heterogeneous (11/21); on postcontrast T1, tumors enhanced homogeneously (14/21) or heterogeneously (7/21); on DWI, tumor ADC values were variable (range 0.8–2.7), suggesting variability in intrinsic tumor properties. *Conclusion*. WB-MRI with quantitative DWI and contrast-enhanced sequences at 3T is feasible and advances the utility of WB-MRI not only to include detection, but also to provide additional metrics for lesion characterization.

## 1. Introduction

 Whole body magnetic resonance imaging (WB-MRI) has been used for tumor detection in several clinical settings, primarily for cancer staging and the detection of metastatic disease (both visceral and bone metastases) [1–8]. A recent application for WB-MRI includes the detection of peripheral nerve sheath tumors (PNSTs) and the assessment of tumor burden in syndromes with a predominance of multifocal PNSTs, such as the NF syndromes, including NF-1, NF-2, and schwannomatosis (SWN) [9–13]. For such applications, a typical WB-MRI protocol includes noncontrast T1 and short tau inversion recovery (STIR), and has thus far been performed utilizing 1.5 Telsa (T) systems with good diagnostic capability.

 However, with the development of new therapeutics there is an increasing demand to measure not only size, but also the biologic properties of these tumors. In particular, functional MRI parameters that include molecular, vascular, and metabolic-based measures are available with diffusion weighted imaging (DWI)/apparent diffusion coefficient (ADC) mapping and dynamic contrast-enhanced MRI and may be applicable to assessing tumor biology longitudinally as well as in response to specific therapies [8, 14]. There are several reports in the literature regarding the feasibility of using “functional” WB-DWI in cancer [3–5, 7, 8, 15–26], but these techniques have not been explored in benign tumors such as PNSTs. 

 The purpose of this study is to show the feasibility of performing functional WB-MRI at 3T inclusive of traditional T1-weighted (T1w) and short tau inversion recovery (STIR) sequences as well as quantitative DWI with ADC mapping and contrast-enhanced T1-weighted sequences. We hypothesized that WB-DWI and WB-contrast-enhanced sequences could be successfully performed in greater than 80% of patients, and offer functional lesional features not provided by standard WB-STIR and T1w, which is important to the characterization of peripheral masses.

## 2. Materials and Methods

### 2.1. Overview

Institutional review board approval and written informed consent were obtained for this prospective HIPAA-compliant study. WB-MRI at 3T including quantitative DWI with ADC mapping and postcontrast T1-weighted imaging was performed in 11 patients who met clinical diagnostic criteria for NF-2 or SWN (Table 1). Image quality (Table 2), a determinant of study feasibility, was reviewed by two investigators, and the presence and character (signal, enhancement characteristics, and ADC values) of peripheral lesions were recorded. 

### 2.2. Subject Population

Patients who had presented for clinical evaluation to a specialty clinic with possible NF-2 or SWN were targeted for recruitment by a neurooncologist. Inclusion criteria were suspected or confirmed NF2 or SWN patients who were referred for WB-MRI for the assessment of tumor burden, no contraindication to MRI, and no prior systemic therapy for NF2 or SWN. Exclusion criteria were contraindications to MRI and prior systemic therapy for NF2 or SWN. 

### 2.3. WB-MRI Technique

**Figure 1:**
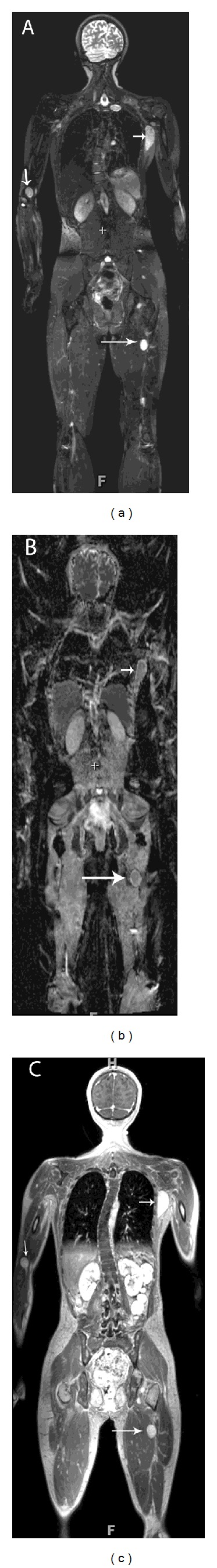
Whole body MRI in a patient with NF-2 and multiple PNSTs, with good diagnostic quality. In (a), whole body STIR (quality ratings of 4 in all body parts except abdomen rating of 3) shows multiple PNSTs in the arm (vertical arrow), chest (short arrow), and thigh (long arrow). In (b), corresponding ADC maping (diagnostic quality of 3 in all body parts except in the arm and neck, where there was a rating of 1 in the arm and a rating of 2 in the chest) shows corresponding PNSTs in the chest (short arrow) and thigh (long arrow) with ADC values of 1.4 and 1.5 × 10^−3^ mm^−2^, respectively. Lesion in the arm cannot be identified on the ADC map due to poor diagnostic quality in the arm. In (c), corresponding T1-weighted postcontrast whole body image shows homogeneous enhancement patterns of the chest, right arm, and thigh PNSTs.

 All studies were performed with a dedicated clinical research 3T scanner (TimTrio, Siemens Medical Systems, Malvern, PA, USA) using the Total Imaging Matrix (TIM). This technology enables the application of multiple phased-array surface coils and receiver channels for parallel imaging with an increased signal-to-noise ratio in three spatial directions while acquiring sections of the body from the head down to the mid femur. This technology requires no repositioning of the patient during the scan. The total scan range of 205 cm was obtained by combining the large field of view (400–500 mm) with automatic table motion. In the coronal plane, four to six areas were scanned. The WB-MRI protocol (shown in Figure 1) consisted of T1-weighted sequences (volume interpolated breath-hold examination (VIBE), TR/TE = 0.88/2/43 ms, field of view = 50 × 50 cm^2^, matrix = 256 × 256, slice thickness = 2 mm), short tau inversion recovery (STIR, TR/TE = 6640/84 ms, field of view = 50 × 50 cm^2^, matrix = 256 × 256, slice thickness = 2 mm with interpolation), and quantitative DWI (TR/TE = 4100/70 ms, *b* = 50, 400, 800 s/mm^2^, field of view = 50 × 50 cm^2^, averages = 4, acceleration factor = 2, slice thickness = 5 mm, bandwidth = 1900 kHz, phase encode lines = 24, echo spacing = 0.64–0.88) with ADC mapping. The acquisition of different *b*-values allowed for the creation of trace ADC maps on a pixel-by-pixel basis for quantitative analysis according to the following equation:

(1) $$\begin{array}{c} \;\text{ADC}=-\overset{n}{\underset{i=1}{\sum }}\frac{\ln\left(S_{i}/S_{0}\right)}{b_{i}}, \end{array}$$

where *b*_*i*_ = the diffusion gradient values, *b* = *γ* ^2^ *G* ^2^ *δ* ^2^(Δ − *δ*/3), *γ* = gyromagnetic ratio, *G* = gradient strength, *δ* = diffusion gradient duration, Δ = time between diffusion gradient pulses, *S*_0_ = 1st image (*b* = 0), and *S*_*i*_ = *i*th image. The DWI sequence *b* value of 50 s/mm^2^ was chosen to reduce perfusion effects [27] and the remaining two *b* values were chosen to obtain an accurate ADC measurement.

**Figure 2:**
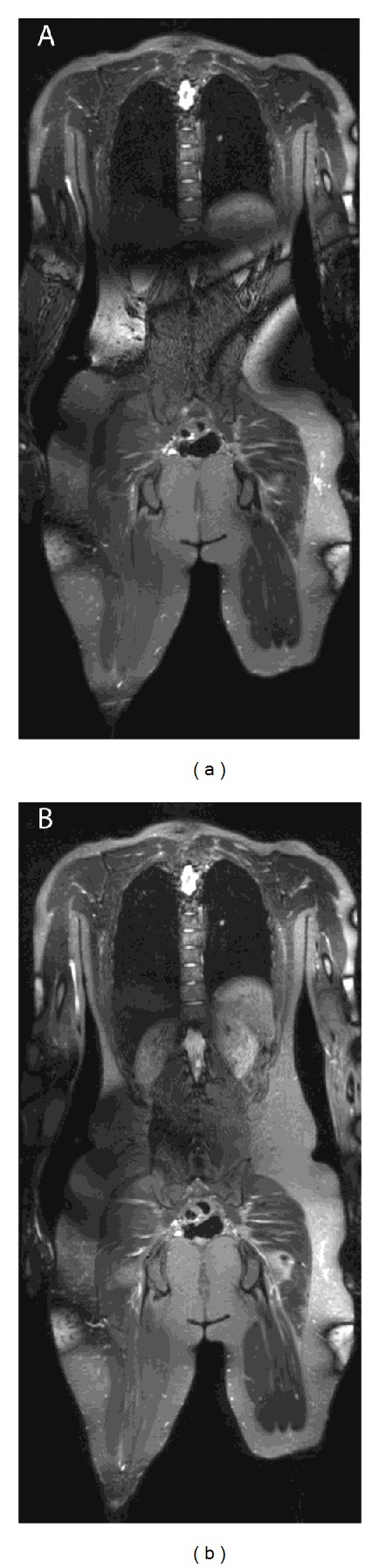
Whole body STIR images in a patient with NF-2 with large artifacts through the abdomen and pelvis, due to inadequate shimming of the large field of view (in (a)) after changing “stations.” In (b), localized fast shimming methods were applied and the shimming was corrected, producing diagnostic quality images.

Subsequently, a postcontrast T1-weighted sequence (VIBE, TR/TE = 0.88/2/43 ms, FOV = 50 × 50 cm^2^, matrix = 256 × 256, slice thickness = 2 mm) was obtained after intravenous administration of 0.1 mmol/kg gadodiamide contrast agent (Magnevist, Bayer Schering Pharma AG, Germany). The contrast agent was injected over 10 seconds with imaging beginning immediately after completion of the injection. The contrast bolus was immediately followed by a 20 cc saline flush. Prior to acquisition of the sequences, whole body shimming was performed. If excessive image noise was identified at the time of acquisition, localized fast shimming was performed as needed.  Figure 2 is an example of the need for localized fast shimming that was performed for improved diagnostic quality. 

All T1-weighted imaging, STIR, and postcontrast T1 imaging were acquired in the coronal plane with isotropic resolution (T1 VIBE) or near-isotropic resolution (STIR), allowing reconstruction into axial and sagittal planes for interpretation. All imaging was respiratory-gated using 2D Prospective Acquisition Correction (PACE) technique to minimize artifacts related to respiratory motion. Total acquisition time was approximately 50 minutes. 

### 2.4. Image Analysis and Reader Procedures

Two readers, one with 10 years experience in musculoskeletal imaging and one with 8 years experience specifically in WB-MRI technique and interpretation, reviewed the images in consensus, (for image quality, lesion detection, and lesion characterization). All imaging planes were reviewed (coronal as well as reconstructed axial and sagittal views). First, readers recorded the diagnostic quality for each study, by body part (chest, abdomen, pelvis, thighs, thoracic spine, lumbar spine, neck, and calves and arms if the latter two were included by imaging). Quality was assessed using a semiquantitative scale, ranging from 1 to 4, with the quality criteria listed in Table 2.

 Second, readers reviewed the imaging and recorded whether peripheral lesions were present or absent, and whether each lesion was visible on every sequence (T1, STIR, DWI/ADC map, and postcontrast T1). STIR images were reviewed first, followed by DWI/ADC maps and then, T1 images with and without contrast. A lesion was defined as any mass-like abnormality of increased STIR signal and was subsequently confirmed on T1-weighted and DWI sequences. Only peripheral lesions greater or equal to 1 cm seen in at least two planes were recorded for the purpose of this analysis. DWI and T1-weighted sequences were also reviewed independently and readers recorded whether additional lesions or incidental abnormalities in other organs were found on these sequences. Readers characterized each peripheral mass by recording whether each lesion met criteria for a PNST (showed evidence of internal enhancement following contrast administration) or a cyst (showed no evidence of enhancement following contrast administration). 

Readers recorded other characteristics of the lesions including the size, location (chest, abdomen, pelvis, thighs, thoracic spine, lumbar spine, neck, calves, or arms), shape (ovoid or irregular), and margin (well-defined, partly defined, ill-defined) of each peripheral lesion. The presence or absence of a target sign, a split fat sign, and perilesional edema were recorded [28]. Signal characteristics relative to muscle on T1-weighted images, STIR and contrast-enhanced imaging (hypointense, isointense, and hyperintense) were recorded along with degree of heterogeneity (homogeneous and less than 25% heterogeneous, moderately heterogeneous with 25%–75% heterogeneity, and markedly heterogeneous with greater than 75% heterogeneity). The presence or absence of associated muscle denervation (increased intramuscular signal on STIR or the presence of atrophy on T1-weighted imaging) was also recorded. The character of the lesion regarding its relationship to the adjacent nerve (eccentric or central) was sought and recorded when possible. For diffusion weighted images; readers assessed the presence or absence of heterogeneity and the presence or absence of the target sign on the ADC maps. Minimum, average, and maximum ADC map values (with standard deviation) were recorded for each peripheral lesion. The latter was accomplished by placement of a region of interest to encompass as much of the whole lesion as possible, with exclusion of surrounding tissues. ADC map measurements were made on one section through the center of the lesion, to avoid volume averaging artifact. Lesions that were less than 1 cm were excluded from the analysis. 

### 2.5. Statistical Analysis

 Descriptive statistics were reported regarding subject demographics, image quality for all body locations, number of lesions, lesion location, and lesion characteristics on the anatomic and functional sequences (signal intensity, heterogeneity, the presence or absence of specific signs described above, and ADC values). 

## 3. Results

Table 3 summarizes the diagnostic quality ratings for each sequence, by body part. Overall, taking all body parts into account, including the spine and neck, diagnostic quality was the highest for T1-weighted sequences (mean 3.95 ± 0.36) and the lowest for DWI (mean 2.53 ± 0.77), across all subjects. For STIR, diagnostic quality was an average of 3.51 ± 0.50, with no quality measurement less than 3. Quality measures were the lowest for the neck and the highest for the abdomen and pelvis, for all sequences. The calves were incompletely included in the field of view of 8 subjects (8/10 patients with NF-2) and the arms were incompletely included in 11 subjects (9/10 patients with NF-2, 1 patient with SWN, and 1 volunteer). An example of shimming artifact that interfered with the quality of the image is shown in Figure 2.

In Table 1, a summary of subject and lesion characteristics is given. A total of 23 lesions (median 3.5 cm, range 1.0–10.2 cm) were identified; no additional lesions were detected on the T1-weighted or DWI sequences if they were not detected on STIR. Lesions were present in the chest (*n* = 7), abdomen (*n* = 4), pelvis (*n* = 4), thighs (*n* = 4), arm (*n* = 1), thoracic spine (*n* = 1), and lumbar spine (*n* = 2). 

Lesion diagnosis and character were determined as follows. On postcontrast T1-weighted images, 21/23 lesions met criteria for a PNST (enhancement following contrast administration), while 2/23 met criteria for a cyst (no evidence of enhancement following contrast administration). Compared to skeletal muscle on T1-weighted images, cysts were all homogeneously hypointense, while PNSTs were homogeneously isointense (*n* = 5/23) or hypointense (*n* = 16/23). On STIR, cysts were homogeneously hyperintense, while PNSTs were all hyperintense but with variable heterogeneity (10 homogeneous, 8 moderately heterogeneous, and 3 markedly heterogeneous). On STIR, the target sign was present in 6/23 cases, the split fat sign was present in 3/23 cases, and perilesional edema was absent in all cases. Muscle denervation was present in 2 cases, in the thigh musculature. In 2/21 tumors, the relationship of the tumor to the adjacent nerve could be resolved and was eccentric to the nerve; in all other tumors, the border with the adjacent nerve was not clearly identified. On postcontrast T1, tumor enhancement was homogeneous in 14/21, moderately heterogeneous in 6/19, and markedly heterogeneous in 1/21.

On diffusion weighted images and ADC maps, 23/23 lesions had diagnostic quality to allow evaluation. All cysts (2/2) were homogeneously hyperintense, while 12/21 PNSTs were homogeneously hyperintense and 9/21 PNSTs were moderately heterogeneous in signal intensity. The target sign was present in 2/21 PNSTs (compared with 6/23 by STIR). For cysts, the mean minimum ADC map value was 2.4 ± 0 × 10^−3^ mm^2^/sec (range 2.4-2.4), the mean average ADC map value was 2.6 ± 0.16 × 10^−3^ mm^2^/sec (range 2.6-2.6) and the mean maximum ADC value was 2.8 ± 0 × 10^−3^ mm^2^/sec (range 2.8-2.8). For PNSTs, the mean minimum ADC map value was 1.36 ± 0.46 × 10^−3^ mm^2^/sec (range 0.8–2.2), the mean average ADC value was 1.89 ± 0.53 × 10^−3^ **mm^2^/sec (range 1.2–2.7), and the mean maximum ADC value was 2.66 ± 0.88 × 10^−3^ mm^2^/sec (range 1.4–3.9).

**Figure 3:**
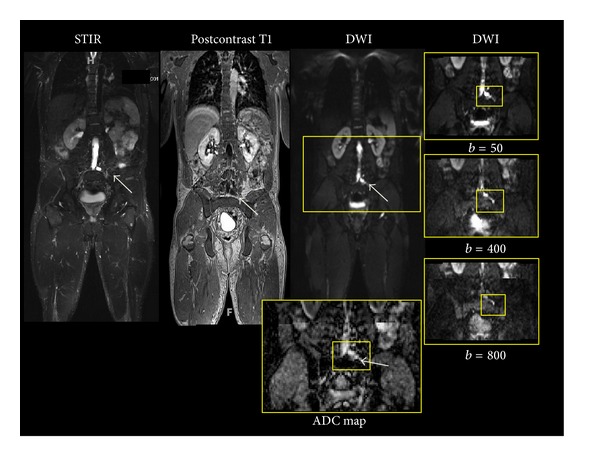
Whole body STIR, postcontrast T1-weighted, and DWI images with ADC map showing a paraspinal cyst (arrows) which is hyperintense on the STIR and DWI images, without demonstrable enhancement on the postcontrast image. The addition of contrast-enhanced sequences to a WB-MRI protocol enables the distinction of cysts from tumors in patients with neurofibromatosis, a distinction not otherwise possible by STIR and T1 imaging alone. In addition, the ADC value of the cyst was 2.6 × 10^−3^ mm^2^/sec in this patient, higher than the peripheral tumors identified in this patient (0.8 and 2.2 × 10^−3^ mm^2^/sec), suggesting that ADC values may provide an additional metric for the distinction of cysts and tumors.

Figure 1 shows the subject in whom the arms were included and a PNST was identified in the arm.  Figure 3 illustrates results in a subject with NF-2 who had peripheral tumors and paraspinal cysts; the cysts could not be distinguished from PNSTs on T1 or STIR but were distinguished by contrast-enhanced T1 and ADC mapping, underscoring the utility of intravenous contrast and DWI for characterizing peripheral lesions. 

## 4. Discussion

WB-MRI with quantitative DWI/ADC mapping and contrast-enhanced sequences at 3T is feasible with high diagnostic quality in patients with PNSTs. The application of this technique to patients with NF2 and SWN is promising, as these additional sequences may advance the utility of WB-MRI not only to include anatomic detection, but also provide quantitative metrics for biologic characterization. The MRI features given by WB-DWI and contrast-enhanced sequences allow for the characterization of tumor cellularity and vascular status.

In previous studies of patients with NF syndromes, WB-MRI was implemented to counteract the need for separate scans of the chest, abdomen, pelvis, and thighs and has been utilized for the detection of peripheral tumors and the determination of disease burden [9, 11–13, 29, 30]. In these studies, WB-MRI was performed at 1.5T and focused on T1 and STIR sequences. Mautner et al. additionally implemented WB-MRI technique with localized contrast-enhanced sequences at 1.5T in NF-1 patients for the assessment of disease burden and identification of malignant peripheral nerve sheath tumors [10]. 

In this study, we assessed the feasibility of expanding WB-MRI techniques to incorporate functional measures with DWI and ADC mapping as well as contrast-enhancement properties. The advantages of performing such studies at 3T include the available increased signal compared with 1.5T, which affords a trade-off for spatial resolution and the ability to perform faster acquisitions with isotropic resolution for the anatomic sequences (VIBE, STIR). Similar acquisitions at 1.5T will require more scanner time and make the scan more susceptible to motion artifacts. In addition, the increased signal available at 3T is theoretically advantageous for acquiring DWI, although susceptibility artifacts may occur with greater frequency at 3T compared with 1.5T [24]. Advances in shimming techniques and the use of a continuous table for WB-MRI, rather than separate station acquisitions, will likely further improve the quality of WB-DWI. In our study subjects, WB-DWI was acquired with acceptable diagnostic quality, although, for most cases, artifact at the edge of the image was present due to the large field of view. For the interpretation of peripheral tumor biology with ADC values in our study, such artifact was not limiting and may be inconsequential to the majority of patients.

Regarding technical factors for performing 3T functional WB-MRI, the coronal plane was chosen for all acquisitions as it inherently combats respiratory motion artifact compared with the axial plane. In addition, the coronal plane is a faster acquisition than the axial plane. Since the anatomic sequence (T1 VIBE, STIR) acquisitions are performed with isotropic or near-isotropic resolution, the datasets can be reconstructed into the axial and sagittal planes (as well as any other oblique plane of interest) with the same resolution as the original acquisition. For detailing the anatomy, our WB-MRI protocol offers good spatial resolution (2 mm slice thickness). For DWI, the acquisition is performed at 5 mm thickness to ensure adequate quality; as DWI is a functional sequence, high spatial resolution is likely less critical. We have demonstrated in this report that peripheral lesions were detectable on the anatomic noncontrast T1, STIR, and postcontrast T1 sequences as well as the DWI sequences and ADC maps. 

Unlike the use of WB-MRI for tumor detection, the addition of contrast-enhanced T1 and DWI sequences enables the characterization of peripheral lesions. First, a common challenge with noncontrast sequences is the differentiation of a tumor from a cyst; with the addition of contrast, the distinction is clear, since tumors enhance with contrast, while cysts do not. DWI offers a theoretical advantage in this regard also, as cysts will likely have a higher ADC map value than tumors given that the ADC is a measure of cellularity. Furthermore, if intravenous contrast is contraindicated or cannot be administered, DWI with ADC mapping can serve as a potentially useful sequence for distinguishing cysts and tumors. Second, the natural behavior of PNSTs in NF2 and SWN is variable between and within patients, with some peripheral tumors having an indolent course and others having a more aggressive course. With the advent of WB-DWI and contrast enhancement, metrics are available which may increase our understanding of tumor biology and the association with clinical behavior in the peripheral tumors of these patients. Interestingly, the contrast enhancement pattern in the subject with SWN (moderately and markedly heterogeneous lesional enhancement) was different from that of the subjects with NF2 (homogeneous lesional enhancement), perhaps reflecting a difference in tumor biology, although longitudinal data is not available on these subjects at this time. Third, the features of peripheral tumors by DWI and contrast-enhanced sequences may ultimately prove useful for determining response to treatment, by assessing changes in the ADC values or contrast-enhancement characteristics following treatment. Currently, only changes in size are used to identify treatment response in the NF syndromes, but WB-DWI has been described for the assessment of treatment response in other disorders such as bone metastases [13, 23, 31, 32], multiple myeloma [33], lymphoma [15, 16], and Rosai-Dorfman disease [34]. In the patients with NF2 and SWN in this study, there was great variability in the ADC values of the peripheral schwannomas, presumably due to the differences in cellularity and relative proportion of Antoni A and Antoni B patterns within each schwannoma. At this time, the utility of WB-DWI and contrast for the assessment of treatment response in the NF syndromes has not been established, as longitudinal studies are needed to determine whether changes in lesional cellularity that may come about following treatment are detectable by alterations in the ADC values or contrast-enhancement pattern of a responding peripheral tumor.

 A limitation of this study is in the use of multistation acquisitions for the performance of WB-MRI, which potentially impacts the quality of shimming and subsequent image acquisition. The multi-station acquisition requires shimming at each station; this has the potential of changing the initial shim values, which may affect the homogeneity of the field. Moreover, the incorrect overlap between each station can lead to “gaps” between major anatomic regions. To overcome these potential problems, the use of an integrating program is needed. Finally, the visualization of WB-MRI data can be a challenge due to the large datasets acquired and methods are being developed to integrate them into one dataset for ease of diagnosis, by creating a multiparametric paradigm in which information from all the sequences is incorporated into one image [35].

## 5. Conclusions

 In conclusion, WB-MRI is feasible for both tumor detection and characterization when applied to the PNSTs seen in patients with NF-2 and SWN. By adding quantitative WB-DWI and contrast to the WB-MRI protocol, metrics for lesion characterization are now accessible and may help increase our comprehension of tumor biology. In turn, WB-MRI may potentially impact clinical outcome by distinguishing the biologically variable PNSTs seen in patients with NF syndromes, potentially impacting decisions for treatment and monitoring response to treatment.

## Figures and Tables

**Table 1:** Subject and lesion characteristics.

Subject	Diagnosis	Lesion diagnosis	Lesion location	Lesion size (cm)	T1w signal	STIR signal	Average ADC (×10^−3^ mm^2^/sec)	Contrast-enhancement pattern
(1)	NF-2	PNST	Chest	2.2	Hypointense	Hyperintense	1.5 ± 0.15	Homogeneous
		PNST	Abdomen	6.9	Hypointense	Hyperintense	1.2 ± 0.17	Homogeneous
(2)	NF-2	PNST	Pelvis	1.2	Hypointense	Hyperintense	1.9 ± 0.13	Homogeneous
		PNST	Pelvis	1.0	Hypointense	Hyperintense	2.2 ± 0.0	Homogeneous
(3)	NF-2	PNST	Pelvis	8.1	Hypointense	Hyperintense	1.4 ± 0.15	Homogeneous
(4)	NF-2	Cyst	Paraspinal/L-spine	2.2	Hypointense	Hyperintense	2.6 ± 0.16	None
		Cyst	Paraspinal/L-spine	1.5	Hypointense	Hyperintense	2.6 ± 0.16	None
		PNST	T-spine	2.6	Isointense	Hyperintense	0.8 ± 0.21	Homogeneous
		PNST	Thigh	2.6	Isointense	Hyperintense	2.2 ± 0.18	Homogeneous
(5)	NF-2	PNST	Chest	2.2	Hypointense	Hyperintense	1.4 ± 0.18	Homogeneous
		PNST	Chest	2.8	Hypointense	Hyperintense	1.2 ± 0.09	Homogeneous
(6)	NF-2	PNST	Chest	10.2	Hypointense	Hyperintense	1.4 ± 0.12	Homogeneous
		PNST	Abdomen	6.4	Hypointense	Hyperintense	1.7 ± 0.22	Homogeneous
		PNST	Arm	3.5	Hypointense	Hyperintense	1.8 ± 0.21	Homogeneous
		PNST	Thigh	4.2	Hypointense	Hyperintense	1.5 ± 0.15	Homogeneous
(7)	SWN	PNST	Chest	8.2	Isointense	Hyperintense	2.7 ± 0.20	Markedly heterogeneous
		PNST	Chest	4.5	Isointense	Hyperintense	2.7 ± 0.40	Moderately heterogeneous
		PNST	Chest	3.3	Isointense	Hyperintense	2.2 ± 0.28	Homogeneous
		PNST	Abdomen	8.2	Hypointense	Hyperintense	1.9 ± 0.55	Moderately heterogeneous
		PNST	Abdomen	8.6	Hypointense	Hyperintense	2.5 ± 0.69	Moderately heterogeneous
		PNST	Pelvis	5.3	Hypointense	Hyperintense	1.9 ± 0.17	Moderately heterogeneous
		PNST	Thigh	8.1	Hypointense	Hyperintense	1.8 ± 0.30	Moderately heterogeneous
		PNST	Thigh	6.5	Hypointense	Hyperintense	2.3 ± 0.37	Moderately heterogeneous
(8)	NF-2	No lesions						
(9)	NF-2	No lesions						
(10)	NF-2	No Lesions						
(11)	NF-2	No lesions						

T1w: T1-weighted imaging; STIR: short tau inversion recovery.

**Table 2:** Criteria for assessing the diagnostic quality of WB-MRI.

Diagnostic quality rating	Criteria
(1)	Nondiagnostic, with artifacts involving greater than 75% of the images
(2)	Limited, with artifacts involving 25%–75% of the images
(3)	Diagnostic, but with artifacts involving less than 25% of the images
(4)	Diagnostic, with no appreciable artifacts

**Table 3:** Average diagnostic quality ratings for each body part and sequence.

Location	T1w	STIR	DWI	T1w + contrast
Spine-chest	3.83	3.59	2.08	3.83
Spine-Abdomen	3.92	3.8	2.92	3.92
Neck	3.92	3.79	1.08	3.92
Chest	4.00	3.56	2.75	4.00
Abdomen	4.00	3.33	3.08	4.00
Pelvis	4.00	3.33	3.08	4.00
Thigh	4.00	3.33	2.83	4.00
Calves	4.0	3.0	3.0	4.0
Arms	3.0	3.0	1.0	3.0
